# Glucose metabolism echoes long-range temporal correlations in the human brain

**DOI:** 10.1162/IMAG.a.1275

**Published:** 2026-06-16

**Authors:** Massimiliano Facca, Anna Ridolfo, Miriam Celli, Claudia Tarricone, Ilaria Mazzonetto, Tommaso Volpi, Andrei G. Vlassenko, Manu S. Goyal, Maurizio Corbetta, Alessandra Bertoldo

**Affiliations:** Padova Neuroscience Center (PNC), University of Padova (Unipd), Padova, Italy; Department of Information Engineering, University of Padova (Unipd), Padova, Italy; Department of Neuroscience, University of Padova (Unipd), Padova, Italy; Venetian Institute of Molecular Medicine (VIMM), Padova, Italy; Department of Radiology and Biomedical Imaging, Yale University, New Haven, CT, United States; Neuroimaging Laboratories Research Center at the Mallinckrodt Institute of Radiology, Washington University School of Medicine, St Louis, MO, United States

**Keywords:** glucose metabolism, fMRI, PET, criticality, long-range temporal correlations, hurst exponent

## Abstract

Intrinsic brain activity is characterized by pervasive long-range temporal correlations. While these scale-invariant dynamics are a fundamental hallmark of brain function, their implications for individual-level metabolic regulation remain poorly understood. Here, we address this gap by integrating resting-state functional Magnetic Resonance Imaging (fMRI) and dynamic [^18^F]FDG Positron Emission Tomography (PET) data acquired from the same cohort of participants. We uncover a systematic relationship between long-range temporal correlations, quantified via the Hurst exponent, and glucose metabolism. Our findings reveal that persistent temporal dependencies are associated with a measurable metabolic cost, with brains exhibiting higher long-range temporal correlations incurring greater energetic demands. Full kinetic modeling of the [^18^F]FDG PET data traces this association specifically to intracellular glucose phosphorylation, pointing to a direct link with neuronal energy metabolism. Beyond glucose metabolism, we also show that these dynamics are likely supported by continuous biosynthetic processes, such as protein synthesis, which are critical for neural circuit maintenance and remodeling. Overall, our results suggest that a significant fraction of the brain’s so-called “Dark Energy” may be linked to spontaneous long-range temporal correlations.

## 1 Introduction

The external environments and internal representations we humans navigate daily are structured across multiple, nested temporal scales. To make sense of the world—whether following a conversation or planning a future action—the brain must bridge milliseconds to minutes, weaving fragmented inputs into a coherent narrative (Baldassano et al., 2017; Hasson et al., 2015). This seamless integration is achieved through multiple processing streams, each operating with its own intrinsic temporal scale, or *Temporal Receptive Window* (TRW, Hasson et al., 2008; Lerner et al., 2011). These timescales, initially compressed in early sensory areas to match the rapid flow of input, progressively expand along the sensory-fugal axis of the brain’s organization (Murray et al., 2014; Raut et al., 2020). At the apex of this hierarchy, TRWs reach their maximal dilation, and activity slowly evolves while keeping track of its previous states—a phenomenon known as *long-range temporal correlations* (Hasson et al., 2008; He, 2014; Linkenkaer-Hansen et al., 2001; Novikov et al., 1997; Raut et al., 2020*)*. Temporal dependencies in neural activity are not only hierarchically organized, but also correlated between task and resting states (Golesorkhi et al., 2021; Ito et al., 2020), suggesting that their spatial arrangement is at least partially hardwired into the brain’s circuitry (Gao et al., 2020). Both computational and empirical studies indicate that highly-connected hubs in the human brain operate at slower timescales (Chaudhuri et al., 2015; Fallon et al., 2020; Gollo et al., 2015), enhancing integration and maintaining an efficient ebb and flow of information across the network—a principle that extends beyond the primate lineage (Sethi et al., 2017). Despite regional differences in intensity and patterning, sluggish temporal dependencies are a ubiquitous feature of the healthy human brain (He, 2011, 2014). From a complex systems perspective, long-range temporal correlations are considered consistent with a system approaching criticality, i.e., the *critical slowing down* (Cocchi et al., 2017; O’Byrne & Jerbi, 2022). Accordingly, in states that push the brain away from the critical point, such as loss of consciousness, these temporal dependencies have been shown to fade, only to rebound with its recovery (Barttfeld et al., 2015; Tagliazucchi et al., 2013, 2016).

Like any other organ, the brain operates at an energetic cost. Its energy budget, however, greatly defies its size: accounting for just 2% of body weight, it consumes approximately 25% of the body’s energy (Clarke & Sokoloff, 1999). [^18^F]Fluorodeoxyglucose Positron Emission Tomography ([^18^F]FDG-PET) is currently the most effective tool to quantify glucose metabolism in the living human brain (Sokoloff et al., 1977). Decades of [^18^F]FDG-PET studies have shown that the metabolic cost of spontaneous brain activity far exceeds that of task-related processing (Attwell & Laughlin, 2001; Raichle & Mintun, 2006). This disproportionate energy use at rest has been metaphorically described as the brain’s “Dark Energy” (Raichle, 2006). To map spontaneous neural activity, resting-state functional Magnetic Resonance Imaging (rs-fMRI) has long stood as the modality of choice, offering a favorable balance of spatial and temporal resolution (Fox & Raichle, 2007; Van den Heuvel & Hulshoff Pol, 2010). Multimodal studies integrating [^18^F]FDG-PET and rs-fMRI have revealed a complex relationship between spontaneous neural activity and glucose metabolism, with evidence suggesting that local activity measures may capture aspects of neuroenergetic coupling that are not fully accounted for by network-level descriptors alone (Aiello et al., 2015; Palombit et al., 2022; Riedl et al., 2014; Volpi et al., 2024, 2025). In a similar vein, metabolic connectivity from functional [^18^F]FDG-PET (fPET) has shown moderate agreement with its hemodynamic counterpart, albeit with stronger links to cognitive functioning (Deery et al., 2025; Voigt et al., 2023). Furthermore, recent rs-fMRI research has shown that many network-level phenomena can be explained by local properties such as temporal autocorrelation, which may ultimately help link neural activity to its underlying biology (Shinn et al., 2023).

**Fig. 1.**
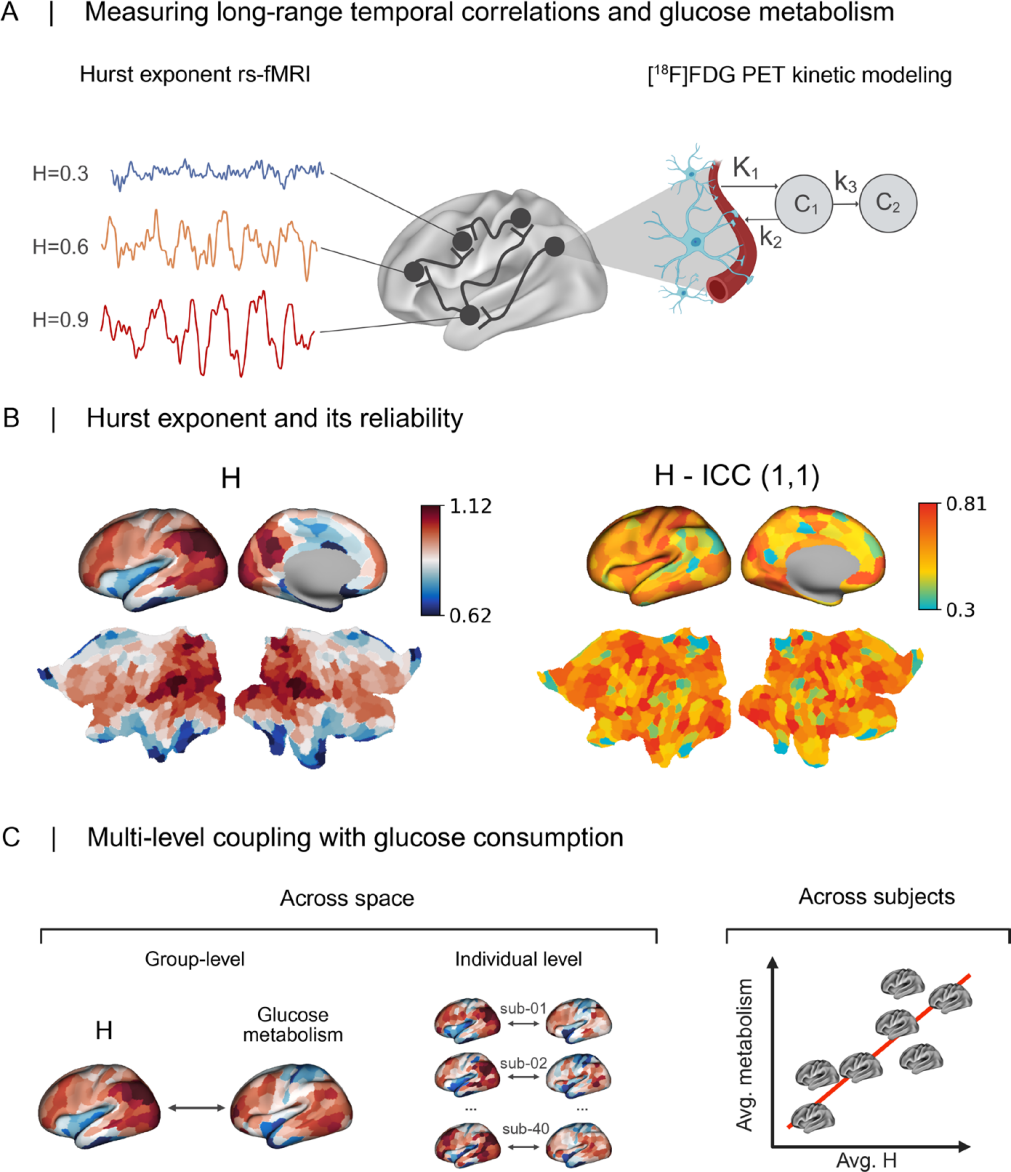
Overview of the study. (A) Long-range temporal correlations are quantified via the Hurst exponent (H) of regional rs-fMRI time series. Representative synthetic time series are shown for increasing values of H, illustrating the transition from antipersistent (H = 0.3) to persistent (H = 0.9) dynamics. Conversely, regional glucose metabolism is measured using dynamic [^18^F]FDG-PET in a quantitative fashion by means of kinetic modeling. (B) Group template map of the Hurst exponent (H). For subcortical structures see Supplementary Figure S1. In the right panel, the intraclass correlation coefficient (ICC-1,1) is shown to highlight the reliability of the Hurst exponent across different rs-fMRI sessions. (C) Overview of the analytical approaches implemented in the present study. The coupling between glucose metabolism and long‑range temporal correlations was investigated both across space, that is, spatial correlations at the group and individual‑subject levels, and across subjects.

Motivated by these findings, here we systematically chart the metabolic correlates of long-range temporal correlations in the human brain. Operationally, we quantify long-range temporal correlations via the Hurst exponent (H), a scalar statistic that measures the degree of temporal dependence in a time series (Hurst, 1951). Higher values reflect more persistent dynamics, whereby the signal retains memory of its past states across multiple timescales. In the context of rs-fMRI, the Hurst exponent has been widely used to characterize scale-invariant neural dynamics (Maxim et al., 2005; Wink et al., 2008) and their sensitivity to state-dependent changes, from task engagement to alterations in consciousness (He, 2014; Tagliazucchi et al., 2016). More recently, it has also emerged as a non-invasive proxy for the synaptic excitation–inhibition (E:I) balance (Trakoshis et al., 2020). To bridge the gap between scale-invariant dynamics and the brain’s energetic profile, our analysis leverages a multimodal dataset comprising rs-fMRI and dynamic [^18^F]FDG-PET (Goyal et al., 2023). By exploiting the full kinetic modeling of [^18^F]FDG in the brain (Sokoloff et al., 1977), we adopt a multilevel perspective (Fig. 1) and show that: (i) regional variability in glucose consumption spatially mirrors regional increases and decreases in long-range temporal correlations at both group and individual levels, and (ii) brains exhibiting stronger long-range temporal correlations incur higher metabolic costs than those with weaker correlations, an effect specifically anchored to intracellular glucose phosphorylation. We also use independent PET atlases to show that the temporal stability of brain activity may be supported by a high rate of protein synthesis and turnover (Schmidt et al., 2005). Collectively, our results point to a potential link between a portion of the brain’s so-called “Dark Energy” and the long-term memory of neural dynamics.

## 2 Methods

### 2.1 Dataset

#### 2.1.1 Research sample

The study cohort consists of 43 healthy subjects (22 female; mean age = 56.2 ± 13.4 years) drawn from the Adult Metabolism & Brain Resilience (AMBR) study (Goyal et al., 2023). Participants underwent a multimodal acquisition protocol comprising MRI and dynamic PET with multiple tracers (see Goyal et al., 2023, for details). MRI and PET scans were conducted sequentially on the same day, except for 3 participants who were scanned on separate days. All imaging procedures were approved by the Human Research Protection Office and the Radioactive Drug Research Committee at Washington University in St. Louis.

#### 2.1.2 MR protocol

MRI data were acquired using a Siemens Magnetom Prisma^fit^ scanner. Each participant underwent a structural MRI scan using a 3D sagittal T1-weighted magnetization-prepared rapid gradient echo (MPRAGE) sequence with 180° radio-frequency pulses (multi-echo: TE = 1.81, 3.6, 5.39, 7.18 ms; TR = 2,500 ms; TI = 1,000 ms; voxel size = 0.8 × 0.8 × 0.8 mm). The average of the first two echoes was used to generate the final T1-weighted image. Following this, two sessions of T2*-weighted gradient-echo echo-planar imaging (GE-EPI) were collected (TR/TE = 800/33 ms, flip angle = 52°, voxel size = 2.4 × 2.4 × 2.4 mm, multiband factor = 6, 375 volumes), including additional spin-echo (SE) acquisitions (TR/TE = 6,000/60 ms, flip angle = 90°) for distortion correction. During the rs-fMRI recording, participants were instructed to keep their eyes closed, remain awake, and refrain from engaging in any directed mental activity.

#### 2.1.3 PET protocol

Each participant underwent a single [^18^F]FDG-PET scan using a Siemens/CTI ECAT EXACT HR+ 962 PET scanner. Dynamic [^18^F]FDG-PET data were acquired for 60 min following the injection of 5.1 ± 0.3 mCi (187.7 ± 12.1 MBq) of [^18^F]FDG. Three 2-mL venous blood samples were collected during the scan to measure plasma radioactivity. Throughout the PET acquisition, participants were asked to rest quietly with eyes closed, maintaining wakefulness and avoiding any structured mental activity. PET data were reconstructed using filtered back-projection with a ramp filter (5 mm FWHM). Attenuation correction was performed using a transmission scan. The dynamic reconstruction consisted of 52 frames of increasing duration: 24 × 5 s, 9 × 20 s, 10 × 1 min, and 9 × 5 min.

### 2.2 Anatomical, rs-fMRI and PET processing

#### 2.2.1 Structural MRI

T1-weighted images were bias-corrected, skull-stripped using SynthStrip (Hoopes et al., 2022), and processed with the standard surface reconstruction pipeline in FreeSurfer (version 7.4.1; Fischl, 2012). All surface reconstructions and segmentations were visually inspected and manually corrected when necessary. Native surfaces were aligned to the Conte69 surfaces using Connectome Workbench tools (Glasser et al., 2013). Registration to MNI space was performed using the deep-learning-based tool SynthMorph (Hoffmann et al., 2022), and the accuracy of each registration was visually checked.

#### 2.2.2 rs-fMRI

Functional data were corrected for slice timing, head motion, and susceptibility-induced distortion using standard tools from the FMRIB Software Library (FSL, Jenkinson et al., 2012). Rigid registration of functional data to the high-resolution T1w was carried out using FreeSurfer’s *bbregister*. Minimally processed data were first projected onto each subject’s native surface and subsequently onto the Conte69 standard surface. Subcortical volumes were nonlinearly warped to 2 mm MNI space using the deformation fields previously estimated with SynthMorph. CIFTI files were generated by combining cortical surface vertices and subcortical voxels. Data were then high-pass filtered (cutoff: 0.008 Hz) and spatially smoothed with a 6 mm FWHM Gaussian kernel. Nuisance regression included the Volterra expansion of the six motion parameters (i.e., 24 regressors), as well as five principal components each from white matter and cerebrospinal fluid signals (aCompCor; Behzadi et al., 2007). Preprocessed data were parcellated using the Schaefer 400 regions 7 Networks atlas (Schaefer et al., 2018), augmented with the Tian subcortical atlas (Version S1, Tian et al., 2020).

#### 2.2.3 Dynamic [^18^F]FDG-PET

Dynamic [^18^F]FDG-PET data were first motion corrected using FSL’s *mcflirt.* Summing the PET frames from the interval between 40 and 60 min post-injection, a PET template map was obtained for registration purposes. Then, in order to obtain the input function for kinetic modeling, motion-corrected PET data underwent a semi-automatic pipeline to extract an image-derived input function (De Francisci et al., 2024). Three late venous samples were utilized to perform the Chen’s spillover correction (Chen et al., 1998). Data were then fitted to the two compartments Sokoloff model (Sokoloff et al., 1977) using a two-step procedure (Volpi et al., 2025). Firstly, k-means clustering was applied to voxel-level PET data to extract 6 Gray matter (GM) and 5 White matter (WM) clusters, on which the Sokoloff’s model was fitted using a weighted nonlinear least squares estimator. Secondly, cluster-wise estimates were propagated at the voxel level using a Variational Bayesian (VB) approach (Castellaro et al., 2017). Thus, for each subject the three parameters describing tracer kinetics were extracted: *K_1_* [mL/cm^3^/min] (influx), *k_2_* [min-1] (efflux), *k_3_* [min-1] (phosphorylation). Based on the three micro-parameters, the net *irreversible tracer uptake* (*K_i_*, [mL/cm^3^/min]) was simply obtained as a linear combination $K_{i}=\frac{K_{1}*k_{3}}{k_{2}+k_{3}}$ at the voxel level. All the estimated parameters were warped to standard space passing through the high-resolution T1w.

### 2.3 Hurst exponent mapping

The Hurst exponent was estimated within a fractionally integrated process (FIP) framework based on the discrete wavelet transform (You et al., 2012). Compared to conventional fractional Gaussian noise (fGn) models, this approach is less sensitive to weak nonstationarities and does not constrain the Hurst exponent to values ≤ 1, thereby allowing for the detection of extended scaling behaviors in neuroimaging data. Estimates with H > 1 are not interpreted within the classical fGn framework, but rather as reflecting strong long-memory behavior under the FIP parameterization. The analysis was implemented using the nonfractal MATLAB toolbox (https://github.com/wonsang/nonfractal), using a univariate maximum likelihood estimation approach with a Haar wavelet filter, and parameter bounds set to lb = [−0.5, 0] and ub = [1.5, 10]. The Haar wavelet was selected for its minimal filter support, which reduces boundary effects in relatively short time series, whereas the selected parameter bounds ensure a broad dynamic range for capturing strong long-memory behavior, thereby avoiding potential ceiling effects, consistent with previous applications to resting-state fMRI data (Fotiadis et al., 2023; Trakoshis et al., 2020). Robustness to filter choice was assessed via a sensitivity analysis; results are reported in the Supplementary Materials (Supplementary Table T3 and Supplementary Fig. S4). Each fMRI run (two per subject) was truncated to the largest dyadic length, retaining N = 256 time points per run (effective duration: 204.8 s), for a total of 512 time points (409.6 s) across both runs. Except for the test–retest reliability analysis (Section 2.5.1), regional Hurst exponent estimates were averaged across the two runs.

### 2.4 Independent datasets

#### 2.4.1 Synaptic density

The atlas is provided by Johansen et al. (2024) and is publicly available online (https://xtra.nru.dk/SV2A-atlas/). Briefly, 33 healthy subjects underwent [^11^C]UCB-J PET, with continuous automatic arterial sampling (first 15 min) followed by manual sampling to obtain a metabolite-corrected arterial input function (AIF). Data were resampled onto standard surface (*fsaverage*) and volumetric (*MNI152*) spaces, followed by spatial smoothing (10 mm FWHM for surface data and 5 mm for volumetric data). Data were fitted with a one-tissue compartment model (1TCM) to obtain tissue distribution volumes (V_T_, [mL/cm³]) for each voxel/vertex. Ex-vivo [³H]UCB-J autoradiography was later used to convert V_T_ values to cerebral SV2A protein densities (B_max_, [pmol/mL]). For full methodological details we refer to the validation paper (Johansen et al., 2024). The atlas in fsaverage space was parcellated according to the Schaefer 400 regions 7 Networks atlas (Schaefer et al., 2018).

#### 2.4.2 Cerebral protein synthesis rate (rCPS)

The atlas is provided by Picchioni et al. (2023) and is publicly available in OpenNeuro (https://doi.org/10.18112/openneuro.ds004733.v1.0.0). In brief, 17 subjects underwent dynamic L-[1-^11^C]Leucine PET and arterial sampling to measure the concentration of labeled and unlabeled leucine in blood plasma. The parameters of the model describing L-[1-^11^C]Leucine kinetics were fitted using a basis function method (BFM) and a voxel-level map of rates of cerebral protein synthesis (rCPS, [nmol/g/min]) was obtained. See the original papers for methodological details (Picchioni et al., 2023; Schmidt et al., 2005; Tomasi et al., 2009; Veronese et al., 2018). The atlas in fsaverage space was parcellated according to the Schaefer 400 regions 7 Networks atlas (Schaefer et al., 2018).

### 2.5 Statistical analyses

#### 2.5.1 Test–retest reliability of the Hurst exponent

Test–retest reliability of the regional Hurst exponent was assessed using the Intraclass Correlation Coefficient under a one-way random effects model with single measurements per session, denoted as ICC(1,1) (McGraw & Wong, 1996). The ICC was computed separately for each brain region across the two rs-fMRI sessions, yielding a regional reliability map. Following established criteria (Cicchetti, 1994), ICC values above 0.40 were considered to reflect fair-to-good reliability. The analysis was performed using the Pingouin library in Python (https://github.com/raphaelvallat/pingouin).

#### 2.5.2 Spatial analyses

To characterize the spatial correspondence between long-range temporal correlations and glucose metabolism, we computed Pearson correlation coefficients between the Hurst exponent and each [^18^F]FDG kinetic parameter at both the group and individual levels. At the group level, correlations were performed between group-averaged maps. At the individual level, spatial correlations were computed across parcels for each participant, yielding a single spatial coupling coefficient per subject, per parameter. Group-level correlations were tested against null distributions generated via the variogram-matching approach of Burt et al. (2020), as implemented in BrainSMASH (https://github.com/murraylab/brainsmash). A set of 10,000 surrogate Hurst maps was generated by spatially permuting the empirical map while preserving its variogram structure. A non-parametric p-value (p_SMASH_) was derived as the proportion of null correlations exceeding the empirical value. The agreement between empirical and surrogate variograms was assessed to confirm the validity of the spatial null model (Supplementary Fig. S2).

#### 2.5.3 Across-subject regression

To assess inter-individual relationships between neural dynamics and metabolic demand, global mean values for the Hurst exponent and each [^18^F]FDG kinetic parameter were computed for each participant by averaging across all brain regions. We then fitted separate linear models with the mean Hurst exponent as the dependent variable and each kinetic parameter as the independent variable. Age, sex, and mean framewise displacement (FD) were included as covariates. Analyses were implemented using Scikit-learn (https://github.com/scikit-learn/scikit-learn).

#### 2.5.4 Multilinear model, dominance analysis, and cross-validation

To decompose the regional variance of the Hurst exponent across distinct aspects of brain biology, we regressed the group-averaged Hurst exponent map on three predictors from independent PET datasets: glucose metabolism (*K_i_,* [mL/cm^3^/min]), synaptic density (Bmax, [pmol/mL], [^11^C]UCB-J), and cerebral protein synthesis rate (rCPS, [nmol/g/min], L-[1-^11^C]Leucine). All variables were z-scored across brain regions prior to model fitting. To rank predictors by their relative contribution, we performed a dominance analysis (Budescu, 1993). Specifically, the Hurst exponent was regressed on all possible subsets of predictors, and general dominance was defined as the average incremental R^2^ contribution of each predictor across all resulting models. Dominance analysis was implemented using netneurotools (https://github.com/netneurolab/netneurotools). The model was cross-validated using the distance-dependent approach of Hansen et al. (2021). Inter-nodal distances were defined as Euclidean distances between region centroids. For each seed region, coefficients were estimated on the 80% of nearest regions and evaluated on the 20% most distant regions. Prediction accuracy was quantified as the Pearson correlation between empirical and predicted Hurst exponent values in training and test sets.

## 3 Results

### 3.1 Long-range temporal correlations in spontaneous brain activity

We first mapped the distribution of the Hurst exponent across the brain using a wavelet-based estimator (see Section 2). The cerebral cortex was partitioned into 400 regions from a well-established functional parcellation (Schaefer et al., 2018), supplemented with 16 subcortical regions (Tian et al., 2020). Long-range temporal correlations were observed throughout the brain: in all subjects and all regions, the Hurst exponent remained above the persistence threshold of H = 0.5. Despite their ubiquity, long-range temporal correlations were not uniformly distributed. The highest values occurred in the precuneus and angular gyrus (Fig. 1B). Because the dataset included multiple rs-fMRI sessions, we quantified the across-session stability of the Hurst exponent using the Intraclass Correlation Coefficient (ICC-1,1; McGraw & Wong, 1996). Following established criteria (Cicchetti, 1994), values greater than 0.4 indicated good reliability. This threshold was exceeded in nearly all brain regions, with a median ICC of 0.61 (MAD = 0.07). These results indicated that the Hurst exponent showed high test–retest reliability across sessions.

### 3.2 The costly architecture of spontaneous long-range temporal correlations

**Fig. 2.**
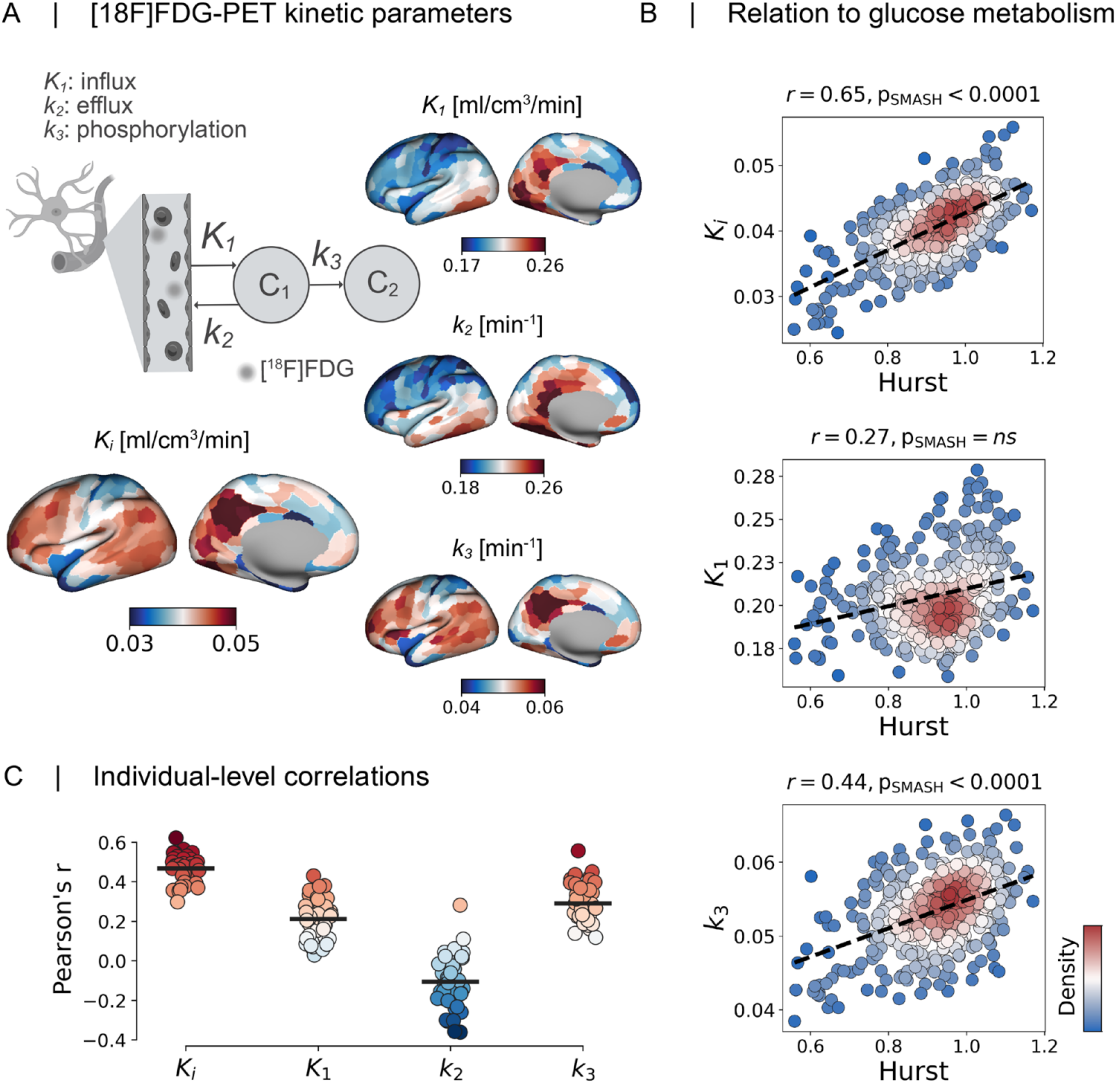
The local cost of long-range temporal correlations. (A) Kinetic parameters describing [^18^F]FDG behavior in the brain: *K_1_* (tracer influx across the blood-brain barrier), *k_2_* (efflux or clearance of [^18^F]FDG into venous blood), and *k_3_* (phosphorylation by hexokinase). Additionally, the macro-parameter *irreversible uptake rate* (*K_i_*) is considered to describe the overall behavior. (B) Group-level associations between the Hurst exponent and [^18^F]FDG kinetic parameters. Long-range temporal correlations were strongly associated with the macro-parameter *K_i_*. Looking at the micro-parameters, the strongest association was found with *k_3_*, i.e., phosphorylation by hexokinase. Correlation with *k_2_* is provided in the Supplementary Figure S3. (C) Individual-level correlations between parameters and the Hurst exponent broadly recapitulate the group-level findings.

Glucose metabolism was inferred from dynamic [^18^F]FDG-PET. For each subject, voxelwise estimates of the kinetic micro-parameters K_1_, k_2_, and k_3_, as well as the macro-parameter K_i_, were obtained (see Section 2). These parameter maps enabled us to assess the spatial association between glucose utilization and the Hurst exponent. For each parameter, we generated a group-level template and quantified its spatial association with the Hurst exponent (Fig. 2A, B), using a spatial-autocorrelation–preserving null model for significance testing (Burt et al., 2020). The group-level distribution of the Hurst exponent was strongly and positively correlated with K_i_ (r = 0.65, p_SMASH_ < 0.0001). Among the micro-parameters, k_3_ showed the strongest association (r = 0.44, p_SMASH_ < 0.0001), whereas K_1_ and k_2_ exhibited no statistically significant relationship with the Hurst exponent (Fig. 2B; Supplementary Fig. S3). At the individual level, the correlational patterns broadly recapitulated the group results (Fig. 2C). Overall, brain regions with stronger long-range temporal correlations utilized more glucose than regions with weaker temporal dependencies.

### 3.3 Long-range temporal correlations and glucose metabolism are coupled across subjects

**Fig. 3.**
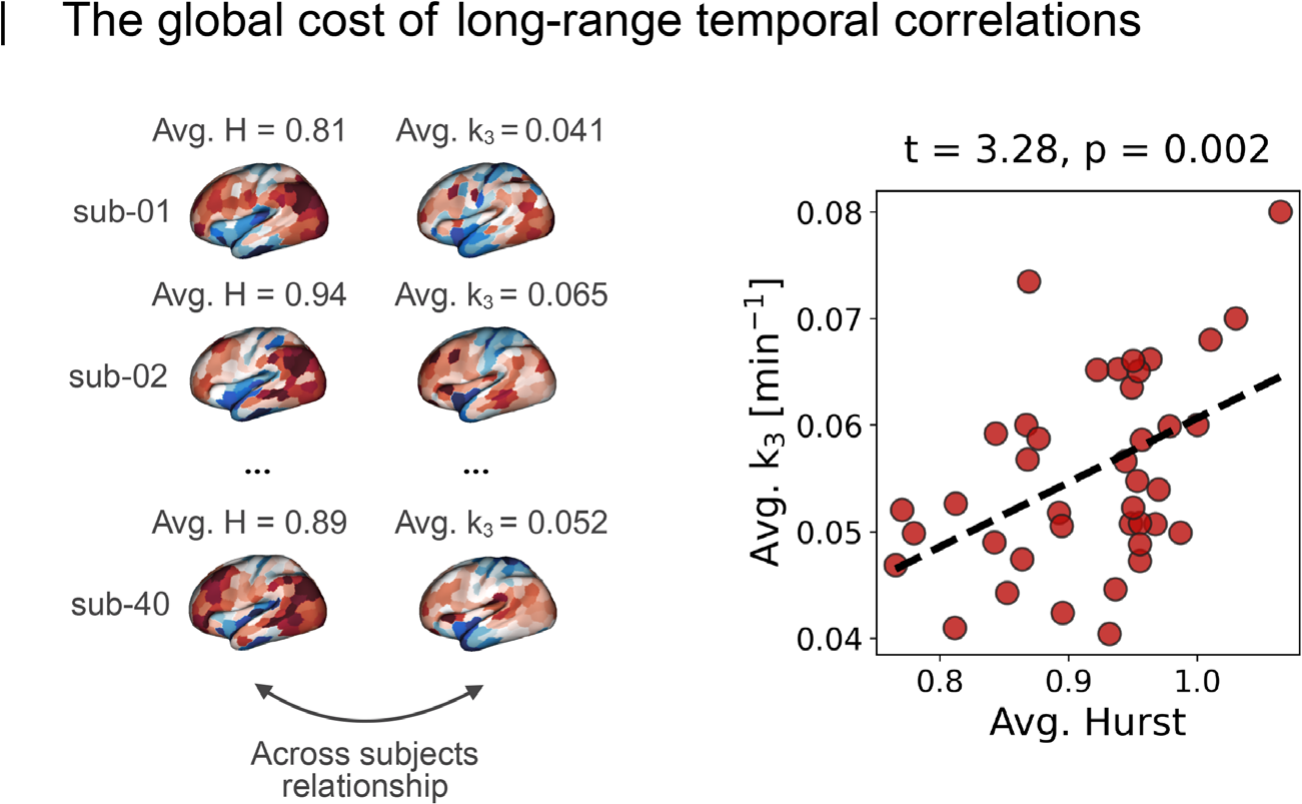
The global cost of persistent temporal dependencies. Across-subject relationship between average Hurst exponent and average k_3_. Each dot represents one subject from the experimental cohort. A higher average Hurst exponent is associated with a faster phosphorylation by hexokinase, that is, higher glucose utilization. The regression model included age, sex, and in-scanner head motion as covariates.

While spatial similarity provided an initial indication of coupling, stronger evidence emerged from across-subject associations. We, therefore, tested the hypothesis that brains with stronger long-range temporal correlations (higher average Hurst exponent) incurred higher energetic demands. To this end, we regressed the average Hurst exponent onto each [^18^F]FDG kinetic parameter, including age, sex, and in-scanner motion as covariates. Across all kinetic parameters, only *k_3_* showed a significant association with the Hurst exponent (standardized β = 0.46, t = 3.28, p = 0.002), whereas no significant effects were observed for *K_1_* (β = −0.0036, t = −0.15, p = 0.90)*, k_2_* (β = −0.22, t = −1.34, p = 0.20), or *K_i_* (β = 0.20, t = 1.31, p = 0.22; see Supplementary Table T1). This result indicated that individuals with stronger long-range temporal correlations exhibited faster phosphorylation by hexokinase, consistent with higher glucose utilization (Fig. 3). Removal of the global signal did not alter this association (R^2^= 0.23; t = 3.46, p = 0.001). Overall, the across-subject analyses supported a relationship between persistent temporal dependencies and glucose metabolism that extended beyond spatial correspondence.

### 3.4 Long-range temporal correlations are associated with both glucose metabolism and protein synthesis

**Fig. 4.**
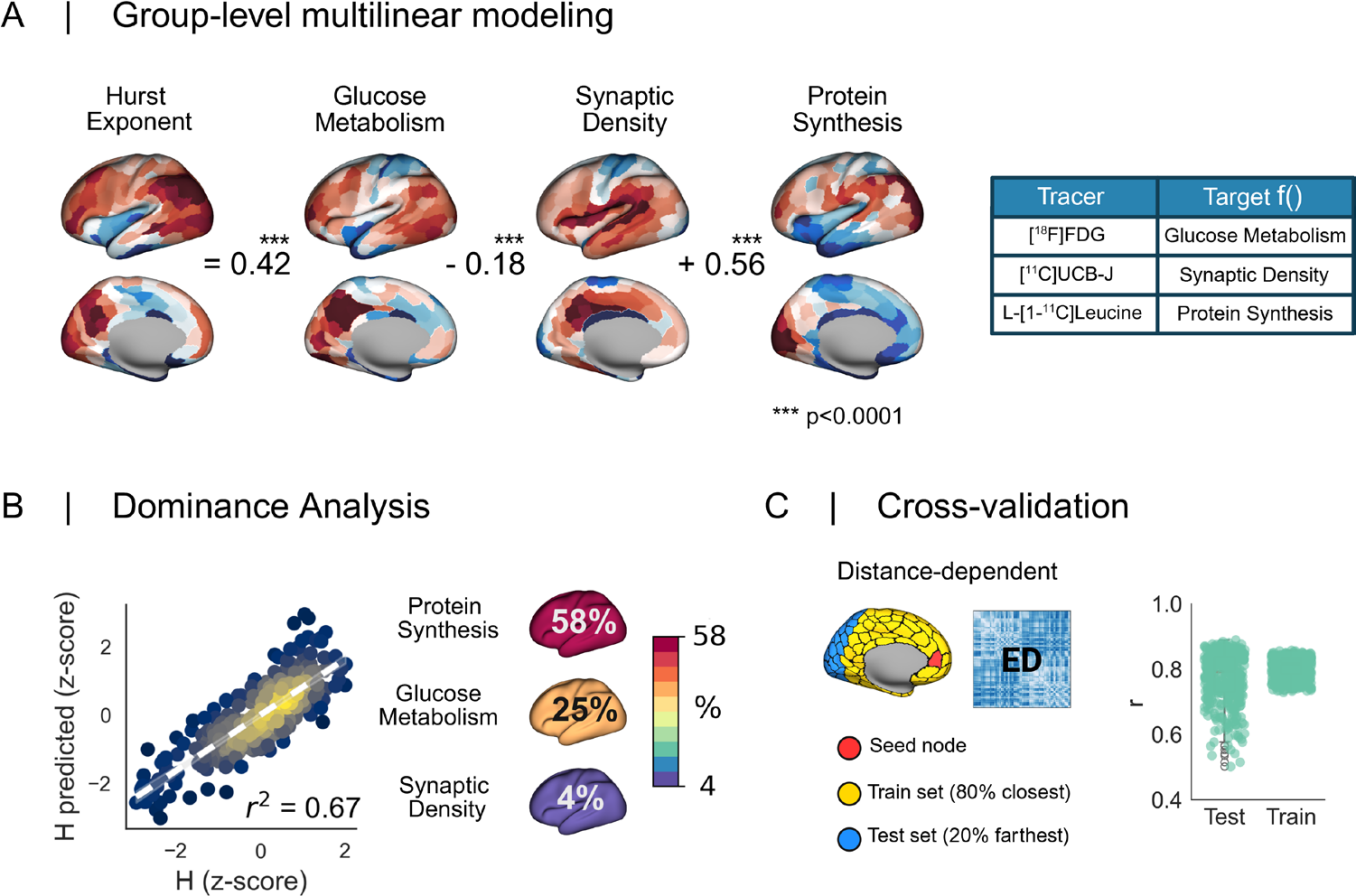
Long-range temporal correlations require not only glucose but also consistent protein synthesis. (A) The group consensus Hurst exponent map is regressed onto three different aspects of brain biology: glucose metabolism, synaptic density, and rate of protein synthesis. The model *R^2^* is greater than that of null models that preserve spatial autocorrelation (p_SMASH_ < 0.0001). The reported beta coefficients are standardized. (B) Dominance analysis for predictor importance. Protein synthesis rate had the highest dominance (58%), followed by glucose metabolism (25%). Synaptic density had little contribution in explaining the regional variation of the Hurst exponent. (C) Distance-dependent cross-validation of the multilinear regression model (Hansen et al., 2021). For each node of the parcellation, the model was trained on 80% of the closest nodes (based on Euclidean distance) and tested on the farthest 20%. Model accuracy was assessed as the correlation coefficient. The average *r* in the test set was 0.70, confirming the validity of the model. Please note that, in this analysis, only cortical ROIs are considered, since the rate of protein synthesis is provided in surface space (see Section 2). ED indicates Euclidean distance.

We previously showed that long-range temporal correlations were associated with higher glucose utilization. We next extended the analysis to two additional biological properties: synaptic density and protein synthesis. This step was motivated by the fact that glucose consumption fuels multiple metabolic processes, including synaptic plasticity. Synaptic density was estimated from [^11^C]UCB-J PET (Johansen et al., 2024), whereas protein synthesis rate was derived from L-[1-^11^C]Leucine PET (Picchioni et al., 2023). These template maps originated from independent datasets. We constructed a multilinear regression model in which the Hurst exponent was regressed onto glucose metabolism (K_i_), synaptic density, and protein synthesis rate (Fig. 4A). The model explained 67% of the variance, and its performance could not be attributed to spatial autocorrelation (p_SMASH_ < 0.0001). Glucose metabolism and protein synthesis showed positive associations with the Hurst exponent, whereas synaptic density showed a weaker negative association. To quantify the relative contribution of each predictor, we performed dominance analysis using general dominance as the metric (Budescu, 1993). Protein synthesis rate exhibited the highest dominance and was therefore the strongest predictor of the Hurst exponent (Fig. 4B). Glucose metabolism was the second-most influential predictor, accounting for 25% of the dominance. After controlling for the other predictors, synaptic density contributed minimally to the regional variation in the Hurst exponent. We then assessed the model’s generalizability. The cross-validation results showed robust out-of-sample performance, with an average correlation of r = 0.70 (SD = 0.02; Fig. 4C). Complementary spatial analyses, in which each FDG microparameter (K_1_, k_2_, k_3_) was included in the multilinear model in place of K_i_, yielded consistent results and are reported in Supplementary Table T2. Taken together, these results indicated that the regional expression of long-range temporal correlations was most strongly associated with protein synthesis, with additional contributions from glucose metabolism.

## 4 Discussion

In this multimodal effort, we sought to untangle the interplay between the brain’s long-range temporal correlations, indexed by the Hurst exponent, and glucose consumption. We exploited a unique dataset comprising rs-fMRI and [^18^F]FDG-PET data acquired in healthy subjects. The multimodal coupling between glucose metabolism and the Hurst exponent was assessed across multiple levels of analysis. We began with group-level associations and progressively incorporated individual-level data, thereby adding nuance to the systematic coupling. Lastly, we contextualized the Hurst exponent in light of two additional PET tracers, [^11^C]UCB-J and L-[1-^11^C]Leucine, used to map synaptic density (Finnema et al., 2016; Johansen et al., 2024) and the rate of protein synthesis (Schmidt et al., 2005), respectively.

Our results show that long-range temporal correlations are a pervasive feature of spontaneous brain activity, consistently observed across regions and individuals. This ubiquity is compatible with the view of scale-invariant neural dynamics as a hallmark of healthy brain function as proposed by the Critical Brain Hypothesis (He, 2014; Hesse & Gross, 2014; Kitzbichler et al., 2009). According to this framework, operating near criticality enables the brain to strike an optimal balance between stability and flexibility, with long-range correlations reflecting the system’s capacity for long-memory processes and efficient information transmission (Cocchi et al., 2017; Expert et al., 2011). Our findings build on this framework by demonstrating a systematic relationship with glucose metabolism: the degree to which brain regions “remember” their past strongly predicts how much glucose they consume as fuel. Crucially, this relationship holds at both the group and individual levels. We then refined our analysis by examining across-subject relationships between the Hurst exponent and metabolic demand. Individuals exhibiting more pronounced long-range temporal correlations consistently displayed accelerated phosphorylation rates of [^18^F]FDG by hexokinase—that is, increased metabolic utilization—as indexed by elevated *k_3_* values. Remarkably, interindividual variability in the Hurst exponent accounted for a significant fraction of the variance in [^18^F]FDG phosphorylation rates across participants. This underscores the central role of intrinsic neural dynamics in shaping the brain’s energetic profile. In essence, brains characterized by more temporally persistent activity patterns are associated with higher metabolic costs, suggesting that the self-similar, scale-free architecture of spontaneous brain activity—while enabling efficient information processing—may exact a significant energetic toll. This finding is consistent with theoretical views suggesting that metabolic resource constraints may act as a control parameter that tunes neural systems toward criticality (Roberts et al., 2014). In this view, the maintenance of scale-invariant dynamics would be intrinsically linked to energy availability, potentially providing an energetic basis for the emergence of long-range temporal correlations.

Beyond glucose consumption, our multimodal analysis reveals that the maintenance of long-range temporal correlations may also be linked to sustained biosynthetic processes. This conclusion stems from the inclusion of a measure of protein synthesis rate in our analysis (Bishu et al., 2008; Hellyer et al., 2017; Schmidt et al., 2005). In particular, rCPS provides a proxy for de novo protein synthesis, a process directly linked to activity-dependent synaptic plasticity and the structural remodeling of neural circuits (Bishu et al., 2008; Schmidt et al., 2005). While the observed spatial correspondence does not imply a direct mechanistic link, it highlights a potential role for biosynthetic turnover in supporting the maintenance of stable neural dynamics over time. The dominance analysis revealed that protein synthesis rate and glucose metabolism jointly accounted for the vast majority of the explainable variance in regional Hurst exponent values. By contrast, synaptic density showed negligible dominance, contributing minimally to the model’s explanatory power. This result is consistent with the presynaptic specificity of [¹¹C]UCB-J: given that SV2A is expressed in presynaptic terminals, this measure is unlikely to capture the postsynaptic processes that account for the dominant fraction of synaptic energy expenditure (Attwell & Laughlin, 2001; Harris et al., 2012), and which are more likely to underlie the metabolic cost of long-range temporal correlations. Taken together, these results suggest that long-memory dynamics are associated with both higher metabolic demand and greater biosynthetic activity, with protein synthesis potentially reflecting the continuous remodeling of synaptic and molecular scaffolds that support functional integration. However, alternative interpretations should also be considered. In particular, regional variations in rCPS may reflect differences in cellular density, baseline transcriptional activity, or broader metabolic turnover, given the close coupling between protein synthesis and these processes. Furthermore, it is important to note that the synaptic density and protein synthesis maps used in this analysis were derived from independent datasets. Future studies acquiring L-[1-¹¹C]Leucine and [¹¹C]UCB-J PET alongside resting-state fMRI within the same individuals will be essential to establish whether the associations reported here generalize at the subject level.

To better understand the origin of this metabolic burden, it is essential to consider the brain’s inherently networked organization (Barabási et al., 2023; Bassett & Sporns, 2017; Bullmore & Sporns, 2009; Sporns et al., 2005). Brain regions do not operate in isolation; rather, they function as parts of distributed, large-scale circuits. These interactions are energetically expensive, yet the associated cost is markedly asymmetric: up to 75% of the brain’s glucose consumption is attributed to postsynaptic activity (Attwell & Laughlin, 2001; Harris et al., 2012; Riedl et al., 2016). This implies that metabolically demanding regions are likely to act as integrative hubs – nodes that receive and process large volumes of afferent input. Strikingly, evidence from the mouse brain shows that such receiver regions, characterized by dense afferent projections, tend to exhibit slower and more persistent temporal dynamics (Sethi et al., 2017). In concert, these findings suggest that the metabolic cost associated with long-range temporal correlations may reflect the integrative demands placed on afferent hubs, which must reconcile multisensory inputs—each with distinct temporal signatures—to support unified brain function (Gollo et al., 2015). From this perspective, the observed energetic footprint likely supports the specialized microcircuitry required to sustain such long-memory dynamics. Whether this cost is driven by the active propagation of signals or by the continuous maintenance of the synaptic scaffolds that enable temporal persistence remains an open question for future research.

### 4.1 Limitations

A fundamental limitation of this study is its observational and cross-sectional nature, which inherently precludes causal or mechanistic conclusions. While the association between glucose metabolism and the Hurst exponent is robust across multiple levels of analysis, our data cannot determine whether metabolic processes drive temporal dynamics or vice versa. Indeed, the observed coupling may reflect shared organizational properties—such as regional cytoarchitecture or microcircuitry—that jointly influence both neural dynamics and metabolic demand, rather than reflecting a direct energetic cost of the temporal correlations themselves. A further limitation concerns the generalizability of our findings. The study cohort primarily represents middle and older adulthood. Both glucose metabolism and long-range temporal correlations undergo substantial changes across the lifespan, including metabolic shifts and changes in the fractal scaling of neural signals (Deery et al., 2024; Dong et al., 2018). Whether the coupling between long-range temporal correlations and glucose metabolism reported here is preserved, attenuated, or reconfigured from early adulthood onward is an open question that future studies in lifespan cohorts will need to address.

Taken together, our results open new avenues for future research. For instance, if long-range temporal correlations are metabolically costly phenomena reflecting the brain’s proximity to criticality, a natural prediction is that conditions disrupting criticality—such as loss of consciousness—should exhibit regional reductions in long-range temporal correlations, accompanied by decreases in metabolic activity. Conversely, we hypothesize that impairments in glucose metabolism through aging, drugs, or disease will have specific effects upon long-range temporal correlations in brain activity. Intriguingly, recent theoretical work suggests that maintaining neural population activity in a “critical” state may help to balance energy costs and computational efficacy (Tatsukawa & Teramae, 2025); future research will be needed to address how this balance is adjudicated at the meso- and macroscopic levels. Further, although our analyses focused on the resting state, future investigations should examine how the coupling between long-range temporal correlations and metabolism reconfigures during task-evoked states. Oxidative phosphorylation is the brain’s primary and most efficient pathway for ATP production (Rolfe & Brown, 1997). However, to accommodate rapid surges in local energy demand, the brain can transiently increase its reliance on aerobic glycolysis—for reasons that remain unclear (Fox et al., 1988; Goyal et al., 2014; Vaishnavi et al., 2010). It is plausible that task-induced modulations of the Hurst exponent may be, at least in part, supported by localized increases in aerobic glycolysis, enabling dynamic adjustments to ongoing computational demands. Unraveling the balance between metabolism and the complex spatiotemporal organization of neural activity represents a compelling path toward understanding the energetic underpinnings of maintaining optimal local and large-scale brain dynamics.

## Supplementary Material

Supplementary Material

## Data Availability

The data are available upon reasonable request. They are not publicly shared to protect the privacy of research participants. The code for estimating the Hurst exponent is publicly available in the MATLAB *nonfractal* toolbox (https://github.com/wonsang/nonfractal). Functions for ICC(1,1) and other versions are available in the Python package Pingouin (https://github.com/raphaelvallat/pingouin). The code for SA-preserving null models is available in the BrainSMASH package (https://github.com/murraylab/brainsmash). The function for dominance analysis is included in the Netneurotools toolbox (https://github.com/netneurolab/netneurotools).
