# Supplementary material for: Glucose metabolism echoes long-range temporal correlations in the human brain
Source: Imaging Neurosci (Camb). 2026 Jun 16;4:IMAG.a.1275. doi: 10.1162/IMAG.a.1275 (PMC13274566; doi:10.1162/IMAG.a.1275)
Supplement: Supplementary Material [file IMAG.a.1275_supp.pdf]

Supplementary Materials

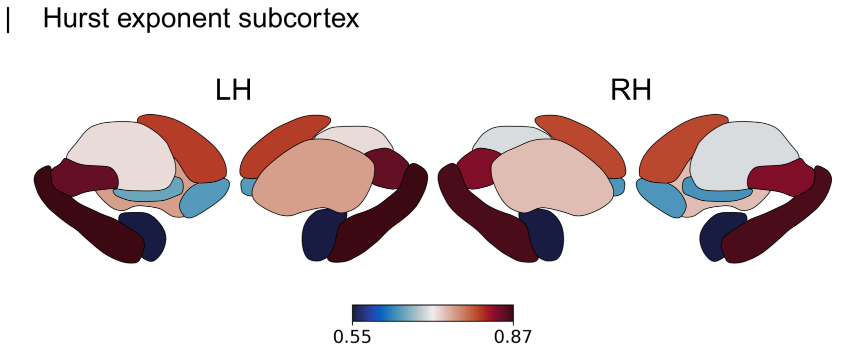

Figure 1: Hurst exponent distribution across subcortical structures. For subcortical data, we used the Melbourne/Tian Subcortical Atlas at the coarser granularity level (S1, 16 regions). As in cortical regions, the Hurst exponent was consistently above the H=0.5 threshold, indicating persistent temporal dynamics. The highest values were observed in the hippocampus, and the lowest in the amygdala. For a complete list of subcortical structures, we refer to the original paper by Tian et al. (2020).

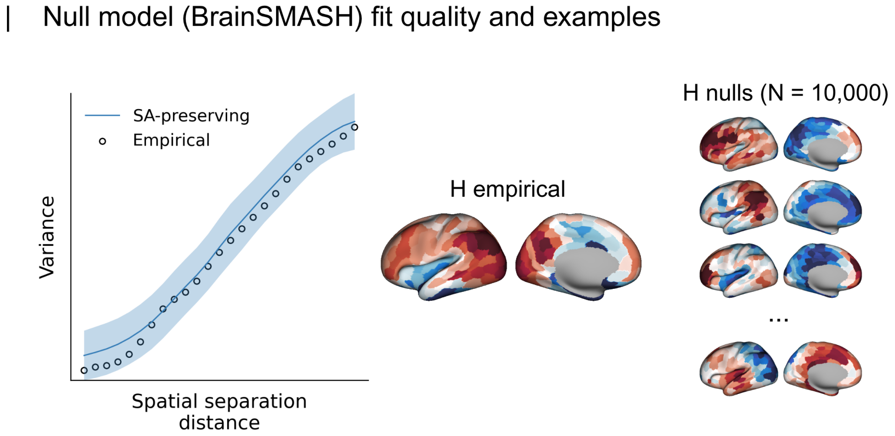

Figure 2: Quality of the brainSMASH fit and examples of null maps. In this work every correlation coefficient was tested against a distribution of null correlations obtained using the BrainSMASH as a null model (Burt et al. 2020). In the leftmost part of the figure the quality of the fit between empirical and surrogate variograms is being shown. In the rightmost part, few null model instances relative to the Hurst exponent map are presented; these maps have the same spatial autocorrelation structure of the empirical one.

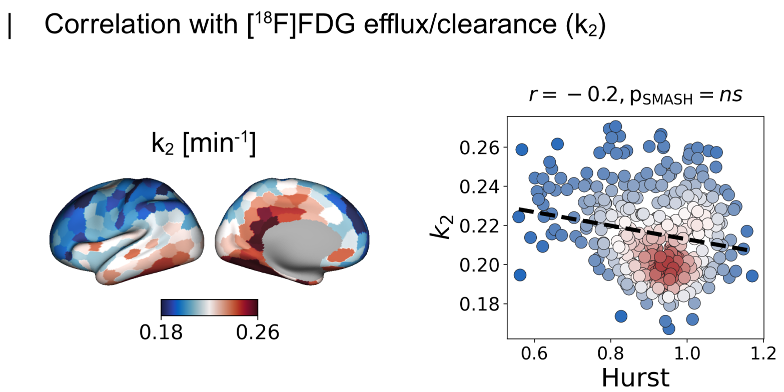

Figure 3: Correlation between Hurst exponent and k_2 at the group level. We observed no significant correlation between Hurst exponent and the [^{18}F]FDG-PET microparameter k_2 [min^{-1}]. This parameter reflects the efflux/clearance of the tracer into venous blood. It is therefore not strictly informative about cell metabolism.

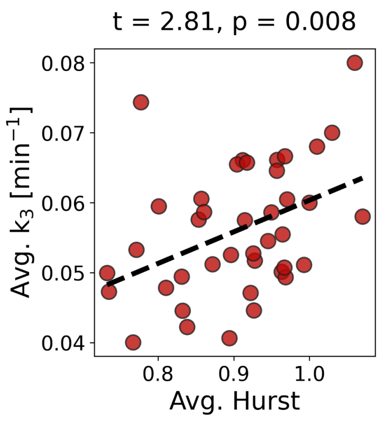

Figure 4: Replication of the across-subject k_3–Hurst relationship. Scatterplot illustrating the relationship between the global average Hurst exponent (estimated using a Daubechies 4 wavelet filter) and the average k_3 across participants. Each dot represents an individual subject. The positive association observed in the main analysis is fully replicated, confirming that the inter-individual association does not depend on the specific wavelet filter used.

	β_{std}	t	p
K_1	-0.0036	-0.15	0.90
k_2	-0.22	-1.34	0.20
k_3	0.46	3.28	0.002
K_i	0.20	1.31	0.22

Table T1. Across-subject associations between the average Hurst exponent and FDG kinetic parameters. Reported values include standardized regression coefficients (β_{std}) and corresponding t-statistics and p-values.

	K_i		K_1		k_2		k_3	
	β_{std}	Dom. (%)	β_{std}	Dom. (%)	β_{std}	Dom. (%)	β_{std}	Dom. (%)
FDG parameter	0.42	25	0.046	5.2	-0.077	4.3	0.311	17.3
Synaptic density	-0.18	4	-0.034	2.1	-0.007	3.5	-0.194	2.6
$rCPS$	0.57	58	0.774	91.7	0.769	92.8	0.724	78.2
R^2	0.67		0.609		0.613		0.665	

Table T2. Multilinear models relating the Hurst exponent to regional glucose metabolism, synaptic density, and protein synthesis. The table reports standardized regression coefficients (β_{std}) and average dominance values for each predictor across four separate models. Each model includes regional synaptic density, protein synthesis rate ($rCPS$), and one of the FDG kinetic parameters (K_i, K_1, k_2, k_3), as predictors of the spatial distribution of the Hurst exponent.

	r	p
K_i	0.66	< 0.0001
K_1	0.27	0.08
k_2	-0.20	0.20
k_3	0.45	< 0.0001

Table T3. Replication of group-level spatial associations using an alternative wavelet filter. The table reports the spatial correlation (r) between the Hurst exponent (estimated using a Daubechies 4 wavelet filter) and each $[^{18}F]$FDG kinetic parameter (K_i, K_1, k_2, k_3). The results demonstrate that the spatial coupling observed in the main analysis is highly robust to the choice of wavelet filter.
